# Pitfalls in quantitative myocardial PET perfusion I: Myocardial partial volume correction

**DOI:** 10.1007/s12350-020-02073-9

**Published:** 2020-02-24

**Authors:** K. Lance Gould, Linh Bui, Danai Kitkungvan, Tinsu Pan, Amanda E. Roby, Tung T. Nguyen, Nils P. Johnson

**Affiliations:** 1 Martin Bucksbaum Distinguished University Chair, Weatherhead P.E.T. Center for Preventing and Reversing Atherosclerosis, McGovern Medical School, University of Texas Health Science Center, Houston, TX USA; 2 Division of Cardiology, McGovern Medical School, UT Health - Houston, Houston, TX USA; 3 Imaging Physics Department, MD Anderson Cancer, University of Texas, Houston, TX USA; 4 Weatherhead PET Center, McGovern Medical School, Houston, TX USA; 5 Programming and Data Management, Weatherhead P.E.T. Center, McGovern Medical School, University of Texas, Houston, TX USA; 6 Weatherhead Distinguished Chair of Heart Disease, Division of Cardiology, McGovern Medical School, Houston, TX USA; 7 Weatherhead PET Center For Preventing and Reversing Atherosclerosis, McGovern Medical School, University of Texas Health Science Center at Houston, 6431 Fannin St., Room MSB 4.256, Houston, TX 77030 USA; 8 Weatherhead PET Center For Preventing and Reversing Atherosclerosis, Division of Cardiology, Department of Medicine, McGovern Medial Medical School, University of Texas, and Memorial Hermann Hospital, Houston, TX USA

**Keywords:** Cardiac positron emission tomography (PET), quantitative myocardial perfusion, coronary flow reserve, partial volume correction, ACR or NEMA PET phantoms

## Abstract

**Background:**

PET quantitative myocardial perfusion requires correction for partial volume loss due to one-dimensional LV wall thickness smaller than scanner resolution.

**Methods:**

We aimed to assess accuracy of risk stratification for death, MI, or revascularization after PET using partial volume corrections derived from two-dimensional ACR and three-dimensional NEMA phantoms for 3987 diagnostic rest–stress perfusion PETs and 187 MACE events. NEMA, ACR, and Tree phantoms were imaged with Rb-82 or F-18 for size-dependent partial volume loss. Perfusion and Coronary Flow Capacity were recalculated using different ACR- and NEMA-derived partial volume corrections compared by Kolmogorov–Smirnov statistics to standard perfusion metrics with established correlations with MACE.

**Results:**

Partial volume corrections based on two-dimensional ACR rods (two equal radii) and three-dimensional NEMA spheres (three equal radii) over estimate partial volume corrections, quantitative perfusion, and Coronary Flow Capacity by 50% to 150% over perfusion metrics with one-dimensional partial volume correction, thereby substantially impairing correct risk stratification.

**Conclusions:**

ACR (2-dimensional) and NEMA (3-dimensional) phantoms overestimate partial volume corrections for 1-dimensional LV wall thickness and myocardial perfusion that are corrected with a simple equation that correlates with MACE for optimal risk stratification applicable to most PET-CT scanners for quantifying myocardial perfusion.

**Electronic supplementary material:**

The online version of this article (10.1007/s12350-020-02073-9) contains supplementary material, which is available to authorized users.

## Introduction

Quantitative myocardial perfusion by positron emission tomography (PET) requires correction for partial volume (PV) loss due to left ventricular (LV) wall thickness being smaller than scanner resolution. Currently, myocardial perfusion by PET is calculated by either of two different perfusion models accounting for PV loss. One model uses time activity curves from arterial and myocardial regions of interest (ROI) on serial, short-duration images fit to a compartmental perfusion model to solve for unknown reconstruction parameters, one of which is PV correction and perfusion; these PV values are not listed explicitly or published but “buried” within flow model equations.^1^,^2^ Consequently, this model is less suited for studying effects of PV corrections on perfusion values and resulting patient management decisions or clinical outcomes addressed here.

Alternatively, a validated “simple” or “retention” perfusion model uses a fixed arterial phase image (2 minutes for Rb-82) followed by a fixed myocardial phase acquisition (5 minutes for Rb-82)^3^-^13^ validated experimentally^3^ as equivalent to fitting time activity curves of a compartmental model for quantitative perfusion and applied clinically (2–16). Equations for the “simple” model use a fixed PV correction determined by imaging phantoms with different size targets from which activity loss is determined as a fraction of known activity.

The one dimension of LV wall thickness is less than PET scanner resolution, whereas LV circumferential and longitudinal dimensions are substantially larger than scanner resolution.^4^ Therefore, the one dimension of LV wall thickness varying through systole and diastole determines the heart rate dependent partial volume loss for quantitative myocardial perfusion by PET^4^ not accounted for by heart models with or without defects. Consequently, we tested the following hypothesis using the retention perfusion model: (A) The two-dimensional American College of Radiology (ACR) phantom rods (two equal radii) or three-dimensional National Electrical Manufacturers Association (NEMA) phantom spheres (three equal radii) to determine myocardial PV loss and corrections overestimate values compared to a one-dimensional reference phantom. (B) Compared to validated low perfusion thresholds associated with ischemia using PV correction derived from one-dimensional limiting wall thickness of the LV,^3^-^13^ overcorrection for PV loss based on ACR or NEMA phantoms results in erroneously high perfusion with consequent impaired risk stratification for MACE, death, or their reduction after revascularization.

## Methods

### Background and Rationale

In cardiac PET, point spread function and loss of peak activity recovery are due to several factors as previously detailed^4^: (i) Limited scanner resolution of 10mm to 20mm full width at half maximum (FWHM); (ii) Positron range; and (iii) Reconstruction parameters and filters.

**Figure 1:**
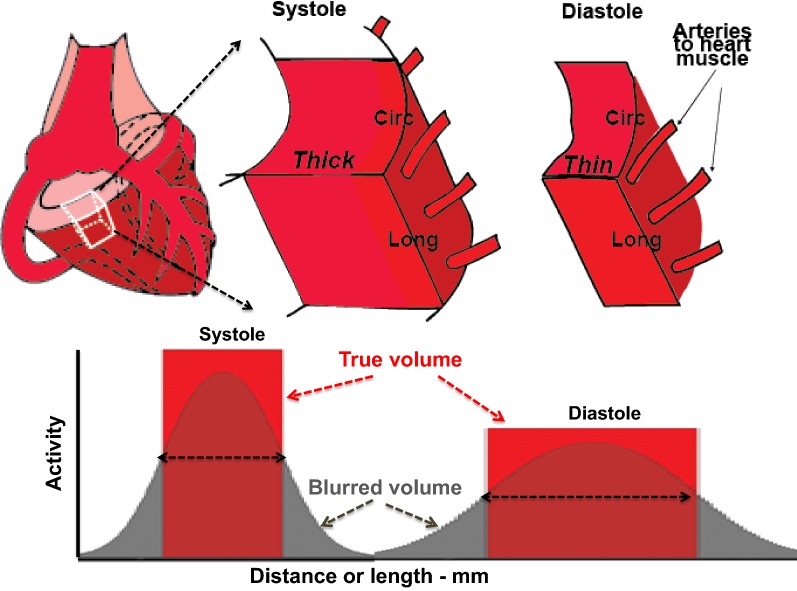
Schema of LV wall with partial volume activity loss due to LV wall thickness during cardiac cycle averaging approximately 15mm, whereas circumferential (Circ) and longitudinal dimensions (Long) are larger than scanner resolution thereby not contributing to partial volume loss

The left ventricle (LV) is a large, tapered cylinder of varying wall thickness during systole and diastole (Figure 1). Circumferential and longitudinal dimensions of LV exceed scanner resolution throughout the heart cycle, thereby engendering negligible PV loss for these dimensions except at the apex. Observed myocardial activity in diastole of 20% to 50% less than in systole is due to diastolic wall thickness less than scanner resolution; thus, myocardial PV loss is due to the *single dimension* of LV wall thickness since circumferential and long axis dimensions are larger than scanner resolution.

PV corrections for cardiac PET are complex for several reasons not accounted for by static, standard phantom measurements and calculations.^4^ The first is failure to account for the unique spatial dimensions of small LV wall thickness with large circumferential and longitudinal dimensions compared to scanner resolution. Secondly, LV wall thickness dynamically changes from systole to diastole with corresponding variable PV loss depending on heart rate.^4^ Accordingly, an approximate average LV wall thickness of 15mm during whole heart cycles as previously reported^4^ was used for estimating one-dimensional PV corrections in patient studies.

This partial volume correction factor is inserted into the equation for calculating cc/min/g for each radial pixel as previously reported^3^-^13^ and in the Online Resource-1 that (i) is highly reproducible ± 10% on test/retest measurement in the same patient within minutes,^5^ (ii) correlates with stress induced angina or ST depression^4^-^13^, and (iii) predicts high risk of death or myocardial infarction^6^,^7^ that is significantly reduced by revascularization.^7^ In order to show their clinical importance, we address the effects on quantitative perfusion and MACE of various PV corrections derived from the one-dimensional tree phantom branches, the two-dimensional ACR rods, and the three-dimensional NEMA spheres.

### Phantom Characteristics

**Figure 2:**
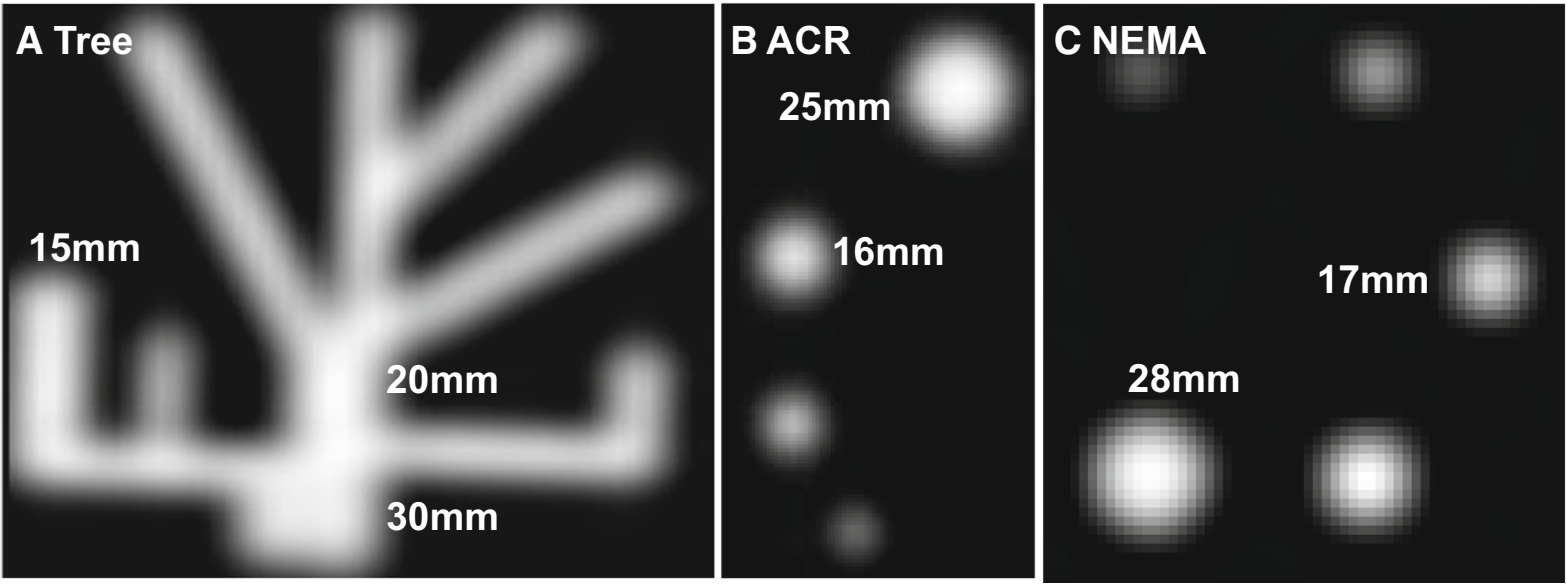
Tomographic images of three phantoms (**A**) One-dimensional tree phantom with branch widths < 20 mm and other dimensions > 20 mm. (**B**) Two-dimensional ACR phantom rods with two equal radii some of which are < 20 mm and rod length > 20 mm. (**C**) Three-dimensional NEMA phantom spheres with three equal radii some of which are < 20 mm

Three phantoms listed below and shown in Figure 2 were filled with approximately 0.37 MBq/mL (10 µCi/mL) of R-82 or F-18 that approximates the myocardial activity observed in our laboratory and imaged by our standard clinical acquisition protocol.^3^-^13^

*NEMA* is comprised of 5 *spheres* of activity each of which is limited in its three dimensions to equal radii of 10 mm, 13 mm, 17 mm, 22 mm, and 28 mm. Therefore, it tests PV loss for a *three-dimensional target*.

*ACR* is comprised of 4 *rods* of activity each of which is limited in its two equal radii of 8 mm, 12 mm, 16 mm, and 25 mm and larger rod length than scanner resolution. Therefore, it tests PV loss for a *two-dimensional target*.

*Tree* phantom developed at the University of Texas has rectangular long and deep dimension > 20 mm with branch *width* being the only limited dimension of 5 mm, 10 mm, 15 mm, 20 mm, and 30 mm. It, therefore, tests PV loss for a *one-dimensional target*.

### Generalized Equation for Cardiac Partial Volume Corrections

Since our Tree phantom is not widely available, we hypothesized that ACR and NEMA phantoms could provide an acceptable approximation of the one-dimensional PV correction using a simple correction (Eq. 1) that accounts for scanner resolution, radionuclide range, and one, two, or three target dimensions less than 20 mm:

1 $$ {\text{PV}}_{\text{observ}} \; = \;\left( {Rx\;{\text{PV}}1{\text{D}}} \right)^{n} $$

where PV_observ_ is measured peak activity recovery of either Rb-82 or F-18 expressed as a relative ratio of the 16 mm/25 mm ACR rods or the 17 mm/28 mm NEMA spheres. Because the cumulative impact of reconstruction algorithms and smoothing filters affects both narrow (16-17 mm) and wide (25-28 mm) targets, it largely cancels out when computing the observed PV ratio. *R* is the activity recovery loss due to Rb-82 positron range relative to F-18. PV1D equals observed activity recovery as the relative activity ratio of the 15 mm/20 mm one-dimensional Tree phantom width for either Rb-82 or F-18, and n is the number of dimensions of each phantom (1 for the Tree, 2 for ACR, and 3 for NEMA).

The reconstruction parameters for every cardiac PET scanner should optimize maximal activity recovery with mild smoothing to reduce statistical noise to acceptable images for clinical interpretation that varies with each facility. At these optimized fixed reconstruction parameters, the one-dimensional PV correction for cardiac PET using Rb-82 or F-18 is derived as the relative activity ratio of the 16mm/25mm ACR rods or of the 17mm/28mm NEMA spheres with the above equation rearranged as follows (Eq. 2):

2 $$ {\text{PV}}1{\text{D}}\; = \;({\text{PV}}_{\text{observ}} /R^{n} )^{1/n} \; = \;^{n} \surd {\text{PV}}_{\text{observ}} /R $$

namely a square root for ACR and cube root for NEMA.

Partial volume correction derived for a phantom is based on “peak activity” recovered for dimensions smaller than scanner resolution. This “peak” partial volume correction must be applied to peak myocardial activity across the LV wall rather than average activity across the wall in order to maintain the principle of preserved area under the activity curve of the point spread function (as the peak of the activity curve decreases, the activity curve widens reflecting increased FWHM). If average activity across the LV wall is used, the partial volume correction needs to be determined by the average activity across the rods of the ACR or spheres of the NEMA phantoms where both are highly variable due tracking borders at statistically poor low counts.

### PET Imaging

As described previously,^3^-^13^ rest–stress myocardial perfusion PET was performed using a Discovery ST PET with 16-slice CT scanner (GE Healthcare, Waukesha, Wisconsin) in 2-dimensional mode with extended septa and reconstruction parameters for theoretical in-plane resolution of 5.9 mm full width at half maximum (FWHM) as defined by NEMA standards with pixel size of 3.27 × 3.27 mm. For patients and all phantoms, images were reconstructed using filtered back projection for *x*-*y* plane with a Butterworth filter for the *z*-axis having a cutoff of 0.52, roll-off of 10 producing uniform comparable activity profiles on short and long axis views of a 20 cm uniformity phantom.

We used standard dipyridamole, adenosine, or regadenoson stress and 1100-1850 mBq (30 to 50 mCi) of generator-produced Rb-82 (Bracco Diagnostics, Princeton, New Jersey) with low-dose CT optimized attenuation co-registration.^4^-^13^

Absolute myocardial perfusion was quantified by HeartSee software (FDA approved 510(k) K171303)^4^-^13^ using arterial inputs personalized for each PET from among five aortic and left atrium locations^4^-^13^ yielding ± 10% test–retest precision within minutes in the same patient.^5^ Regional rest and stress flow (cc/min/g) and CFR as stress/rest ratio were determined for each of 1344 pixels in the LV. CFC integrates regional pixel values of stress cc/min/g and CFR into 5 color ranges from red (normal, healthy volunteers) to blue (severely reduced with angina or ST changes during stress) as previously reported^4^-^13^ and detailed in the Online Resource figure.

Subjects undergoing diagnostic PET for quantitative myocardial perfusion since mid 2007 were analyzed as previously reported.^4^-^13^ All subjects were followed for MACE including all-cause death, non-fatal myocardial infarction, and revascularization after the PET.^6^,^7^ All subjects signed written informed consent approved by the University of Texas Committee for the Protection of Human Subjects. Quantitative rest/stress PET perfusion and following up for events after PET were obtained from 3987 cases of which 3800 had no MACE and 187 had MACE.

In the absence of an absolute “gold standard” for myocardial activity or perfusion, we used as a “reference standard” the MACE outcomes (death, MI, or revascularization) with and without different PV corrections to demonstrate their importance on clinical management. For 3987 PETs, rest perfusion, stress perfusion, and Coronary Flow Capacity (CFC) per pixel were recalculated for each PET using PV corrections of Table 1 for the ACR 16mm two-dimensional rods and the NEMA 17mm three-dimensional spheres. Cases were divided into 187 PETs followed by MACE after PET and 3800 without MACE in order to demonstrate the impact of PV corrections changing perfusion and CFC sufficiently to impact risk stratification for known MACE.

**Table 1:** PV loss for 2D GE DST PET-CT with F-18 and Rb-82 in Tree, ACR, NEMA phantoms

Phantom	Tree one dimension 15mm	ACR two dimensions 16mm	NEMA 3 dimensions 17mm
F-18 PV loss	0.94	0.85	0.72
Rb-82 PV loss	0.90	0.73	0.59

### Statistical Analysis

Mean ± standard deviations are reported for continuous variables, number with percent for categorical variables, and mean with one standard deviation for continuous variables with skewed distribution. We utilized paired or unpaired t tests to compare continuous variables and Chi-square or Fisher’s exact test to compare categorical data. Kolmogorov–Smirnov (KS) tests compared histogram distributions between groups in color-coded ranges of relative regional uptake images and regional CFC distribution of the left ventricle.^5^

## Results

In Figure 2, spreading effects and PV loss cumulatively from limited resolution, positron range, and reconstruction parameters-filters increase with each additional dimension of target activity less than 20mm. Therefore, peak recovered activity decreases with each additional target dimension less than 20mm since the total cumulative activity under one-, two- or three-dimensional area, or volume plots of relative activity has to remain constant for fixed activity in the phantom target as also illustrated in Figure 1.^4^

Table 1 shows for F-18 and Rb-82, the progressive PV loss of activity from the one-dimensional tree phantom to two-dimensional ACR phantom to three-dimensional NEMA phantom. In addition, PV loss for the 15mm one-dimensional Tree phantom and the16mm two-dimensional ACR rods are comparable for Rb-82 and F-18. However, for each target size within the ACR and NEMA phantoms, the PV loss for Rb-82 is greater than for F-18 due to greater positron range.

**Figure 3:**
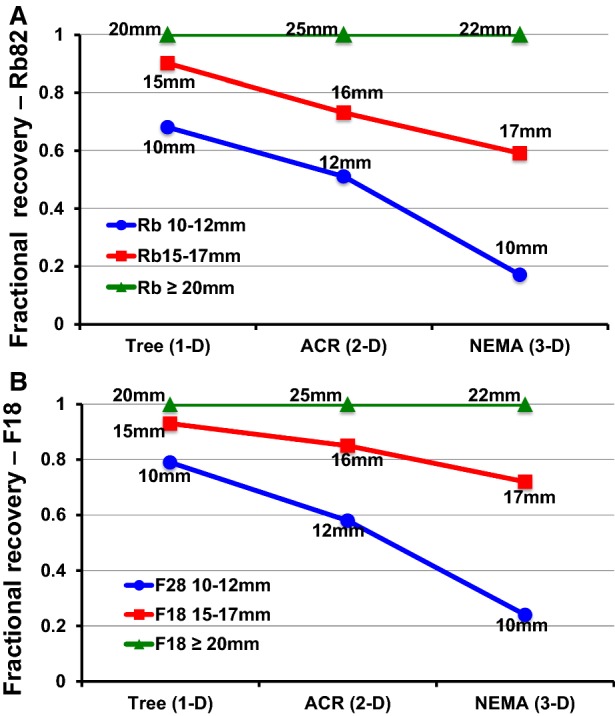
Plots of fractional relative activity recovery for approximately comparable dimensions of three phantoms for Rb-82 (**A**) and F-18 (**B**)

Figure 3 plots relative activity recovery for Rb-82 (A) and F-18 (B) for approximately comparable size targets of the one-dimensional Tree branch, two-dimensional ACR rod diameters, and three-dimensional NEMA sphere diameters, respectively, as follows in corresponding order of Tree, ACR, NEMA: 10-12-10 mm, 15-16-15 mm, and 20-25-30 mm. The largest sizes > 20 mm show equal activity recovery as the denominator reference for the smaller dimensions for which activity is expressed as a relative ratio to the 20 mm or greater target size.

Table 1 and Figure 3 demonstrate an important concept for PV correction. For optimal reconstruction parameters, the resolution and PV loss of a scanner are fixed values characteristic of that scanner, isotope, and reconstruction filters as measured by ACR or NEMA phantoms. However, for the same reconstruction parameters, Table 1 and Figure 3 also show that for target sizes less than 20 mm, activity recovery and PV loss depend on target size and shape, i.e., whether there are one, two, or three target dimensions. Therefore, PV correction depends on target shape and size separately from and in addition to all the other factors. The aortic and left atrial target sizes for arterial input are usually over 20mm and, therefore, do not require PV correction.

In Table 2, PV correction based on PV loss observed for the 16mm diameter two-dimensional rods of the ACR phantom overestimates the correction for one-dimensional LV wall thickness. Similarly, PV correction based on PV loss observed for the 17mm diameter three-dimensional spheres of the NEMA phantom substantially overestimates correction for one-dimensional LV wall thickness. Both ACR and NEMA phantom data overestimated PV corrections for LV causing erroneously high myocardial perfusion with consequent impaired risk stratification unrelated to validated low flow thresholds of perfusion associated with ischemia, MACE, or death and their reduction after revascularization.^7^,^8^

**Table 2:** Rest and stress perfusion, CFR with partial volume corrections based on 1D, 2D, and 3D phantoms (N = 186) using Rb-82

PET Metric	Tree 1D stand PVC 0.9	ACR 2D PVC 0.73	NEMA 3D PVC 0.59
Rest cc/min/g	0.78±0.07	1.20±0.16	1.81±0.33
Stress cc/min/g	1.35±0.22	2.00±0.42	2.89±0.76
CFR	1.82±0.46	1.78±0.43	1.70±0.36

Paired T test between all columns for each row P < 0.000001

*PVC*, Partial volume correction; *CFR*, coronary flow reserve

Tables 1, 2 and Figure 3 show that the relative activity ratio of two-dimensional ACR 16 mm/25 mm rods imaged with Rb-82 overestimates the one-dimensional PV correction perfusion incorporating the positron range of Rb-82. However, due to the shorter positron range of F-18, the PV correction based on the ACR 16 mm/25 mm rods imaged with F-18 is less than for Rb-82 and largely reduces the overestimated PV correction of the ACR phantom imaged with Rb-82. Therefore, from a practical viewpoint for Rb-82, the PV correction based on the ACR 16/25 mm rods using F-18 approximates the PV correction derived from the one-dimensional Tree 15 mm/20 mm width using Rb-82.

### Systematic Impact of Erroneous LV Partial Volume Corrections on Quantitative Perfusion, Risk Stratification, and MACE

**Figure 4:**
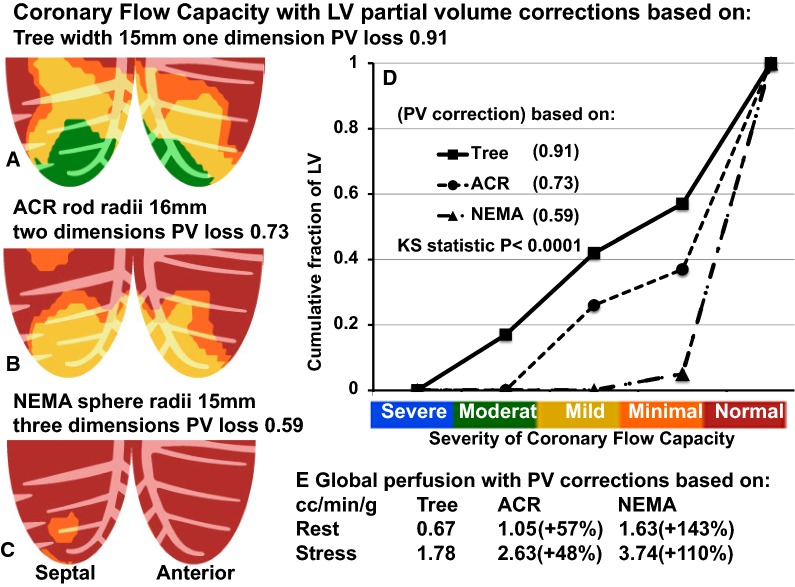
Coronary Flow Capacity for one PET case with perfusion and CFC map determined using (**A**) standard one-dimensional PV correction (tree phantom) compared to using PV corrections derived from ACR (**B**) and NEMA (**C**) phantoms with Kolmogorov–Smirnov cumulative histogram plots and statistic (**D**). PV corrections derived from ACR and NEMA phantomst are expressed as percent increase over the one-dimensional PV correction derived from the tree phantom (**E**)

As an example of a patient with CAD undergoing quantitative PET with Rb-82, Figure 4 shows septal and anterior views of abnormal Coronary Flow Capacity (CFC) due to LAD occlusion. For this case, the CFC severity histogram plots and Kolmogorov–Smirnov (KS) statistic compare the cumulative fractional distribution of CFC severity for the entire LV using different PV corrections from Tree, ACR, and NEMA phantoms for quantitative perfusion. For each separate CFC map, its cumulative CFC histogram distribution and its KS statistic uses one-dimensional PV correction derived from the 15 mm Tree phantom (A) or two-dimensional PV correction derived from the 16 mm ACR rod (B), or three-dimensional PV correction derived from the 17mm NEMA sphere (C).

In Figure 4, PV corrections derived from Tree, ACR, and NEMA phantoms progressively increase average global resting and stress perfusion by 50% to 150% toward normal or high values that eliminate significant CFC abnormalities despite a known occluded coronary artery. The progressively larger PV corrections derived from Tree to ACR to NEMA phantoms progressively shifts the CFC cumulative severity histogram downward and rightward into erroneously high values within the range of normal PETs.

**Figure 5:**
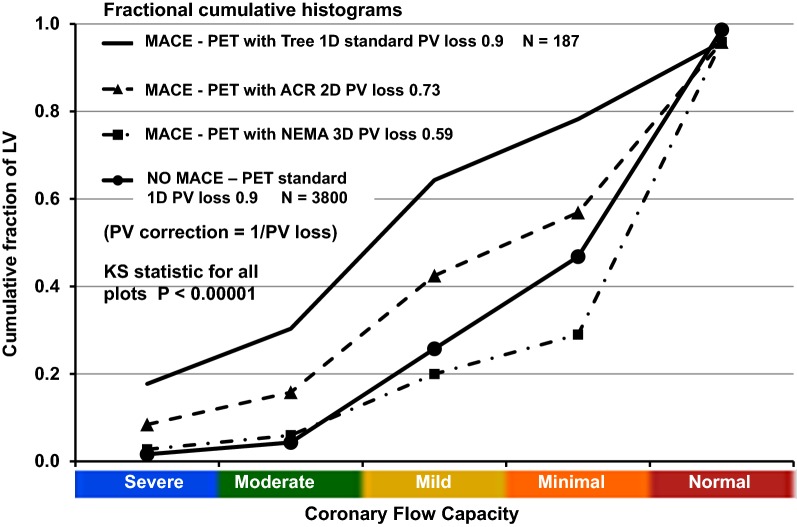
Changes in Coronary Flow Capacity (CFC) due to PV corrections derived from the 1D tree phantom, the 2D ACR rods, and 3D NEMA spheres using MACE over 10-year follow-up as the outcome defined reference of severity for 3987 PET cases without (3800) and with MACE after PET (187). The cumulative histograms and Kolmogorov–Smirnov statistic for CFC of the MACE group based on perfusion using standard one-dimensional PV correction of the tree phantom (solid line) is higher and leftward indicating much worse CFC size-severity compared to the no MACE group (solid line with circles). For larger PV corrections derived from two-dimensional ACR rods (triangles with dashed line) and three-dimensional NEMA spheres (squares with dash dot line), histogram size-severity distribution of PET with MACE shifts downwards and rightwards towards the normal range for PETs with no MACE for PV corrections based on ACR (dashed line with triangles) or NEMA phantoms (dashed-dot line with squares line with triangles) that are uncorrected to 1D PV by our simple formula. For the NO MACE group with PV corrections based on 2D ACR or 3D NEMA PV loss, the CFC size-severity histogram distributions as % of LV are also shifted downward and rightward toward larger % of LV in the high flow red (normal) or orange (minimally reduced) range and hence of no clinical consequence compared to the 1D PV corrections for the MACE group, hence not shown

Figure 5 shows the resulting rest–stress perfusion and CFC cumulative histograms and Kolmogorov–Smirnov statistic comparing histogram distributions of CFC severities based on the ACR or NEMA phantom PV corrections for the PETs without and with subsequent MACE.

The progressively larger PV corrections derived from Tree to ACR to NEMA phantoms progressively shifts the CFC cumulative severity histogram downward and rightward into erroneously high values toward or within normal ranges. The resulting high perfusion levels are well above the CFC threshold associated with clinical ischemia (4–16) and high risk of death or MI^6^,^7^ that are reduced by revascularization.^7^ Accordingly, the overestimated PV corrections and overestimated perfusion values derived from ACR and NEMA phantoms may impair accurate risk stratification and hence potential coronary interventions guided by perfusion severity.

### Validation of PV Correction Equation from ACR and NEMA Phantoms

As demonstrated in Table 1, *R* is the activity recovery loss due to Rb-82 positron range relative to F-18 that is 0.96 for Rb-82 (0.90/0.94 in Table 1) and 1.0 for F-18.

For an observed relative activity recovery of 0.85 from the 16/25mm ACR rods, the PV correction for the one-dimensional PV correction for LV imaging with F-18 is calculated as

PVD1 = (0.85/1)^1/2^ or ^2^√0.85 = 0.92 that compares with the directly measured activity recovery of 0.94 for F-18 from the 15 mm/20 mm activity ratio of the Tree width in Table 1.

For cardiac Rb-82 imaging, PV activity recovery for the same observed activity recovery of F-18 in the ACR and NEMA phantoms would be as follows. For an observed activity recovery of 0.73 from the 16 mm/25 mm ACR spheres, the PV correction for the one-dimensional PV correction for cardiac imaging with Rb is calculated as: PVD1 = (0.73/0.96^2^)^1/2^ or ^2^√0.79 = 0.89 that compares with the directly measured activity recovery of 0.9 for Rb from the 15mm/20mm relative activity ratio of the Tree width in Table 1. The Online Resource-1 has additional examples of this equation for the NEMA phantom.

### Advanced, High-Resolution 3D PET-CT Scanners—Progressive Partial Volume Loss with Limited Size of 1 Dimension (Tree Phantom), 2 Dimensions (ACR), and 3 Dimensions (NEMA)

**Fig. 6:**
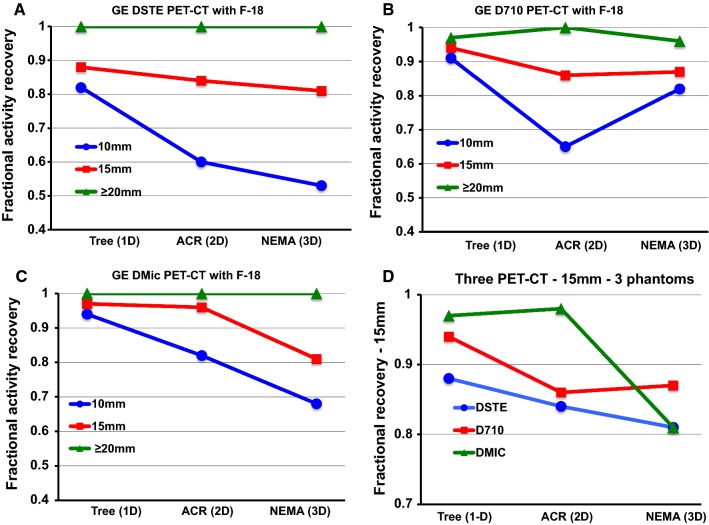
Fractional relative activity recovery for GE DSTE (**A**), D710 (**B**), DMic (**C**), and for three 3D PET-CT scanners (**D**) for 10mm, 15mm, and ≥ 20mm of one-dimensional tree phantom, two-dimensional ACR rods, and three-dimensional NEMA spheres filled with F-18

Figure 6 shows PV corrections for F-18 in the ACR phantom for three different advanced, high-resolution, 2D (GE DSTE)(A) and 3D PET-CT scanners, GE D710 (B), and DMic (C). For comparison among these scanners (D), relative activity recovery from different sized phantom targets was extrapolated to match the 10 mm, 15 mm, and 20 mm sizes of the tree phantom for simplified correct comparative plots in Figure 6D. All scanners show the same trend as the older Discovery ST PET with 16-slice CT with decreasing activity recovery for the same size targets of the one-dimensional Tree phantom (branch width) to two-dimensional ACR rods (two equal radii) to three-dimensional NEMA spheres (three equal radii). This observation suggests a general principle for cardiac PET-CT scanners for the one-dimensional PV loss related to LV wall thickness where the partial volume correction value is specific and needs determining for each different type of scanner using one of these phantoms.

## Discussion

For quantitative activity recovery by PET, our phantom data confirm a basic principle that PV loss of peak activity progressively declines with each added target dimension smaller than 20 mm. Consequently, quantifying myocardial perfusion requires a one-dimensional PV correction for the single dimension of LV wall thickness since circumferential and longitudinal dimensions are greater than scanner resolution with no significant PV loss in those dimensions. Since ACR rods have two dimensions (two equal radii) and NEMA spheres have three dimensions (three equal radii) less than 20 mm, partial volume corrections based on these phantoms need to be corrected to the equivalent of a one-dimensional target by an equation as proposed and validated in this manuscript. As a simple practical step, the optimal LV myocardial PV loss and correction for Rb-82 and F-18 can be derived from the widely available ACR phantom filled with F-18 as the relative activity ratio of the 16 mm/25 mm diameter rods.

### Limitations

PV loss and corrections vary with systolic–diastolic LV wall thickness and hence with heart rate since tachycardia during pharmacologic stress systole increases cumulative systolic time. However, time-varying PV corrections during diastole and systole proved complexly impractical^4^ and substantially less important than the correct, fixed, one-dimensional PV correction for an approximate fixed LV wall thickness of 15 mm.

## Comparison to the Literature

Commonly, PV corrections may be determined by compartmental flow modeling with PV correction as one of several unknowns whose value is solved by curve fitting to time activity curves.^1^,^2^ This method assumes very high fidelity, correct, time activity curves within a fixed ROI on the LV. However, the values of these PV corrections are not listed or published but “buried” within the flow model equations.^1^,^2^ Consequently, this model is less suited for studying effects of variable partial volume corrections on perfusion values defining regional physiologic CAD severity to guide management or related to MACE.

Moreover, time activity curves from this sample volume may be corrupted by cardiac motion, translation, and wall thickening within a varying attenuation environment as the heart commonly moves up to 20 mm vertically and medially superimposed on respiratory positional changes of the heart, mediastinum and surrounding attenuation structures.^4^,^5^,^8^-^10^ In addition, as the sample volume for time activity curves decreases in size from an entire arterial distribution down to secondary branches, to one cm^3^ or one pixel, the time activity curves are increasingly subject to random count variability (statistical noise) thereby limiting reliability of regional time activity curves for regional quantitative perfusion of actual arterial distributions.

## New Knowledge Gained

For quantifying myocardial perfusion by PET to predict high-risk CAD that is significantly reduced by revascularization, partial volume loss due to LV wall thickness less than scanner resolution can be corrected by cardiac specific adaptation of phantoms with limited size in 1 dimension (Tree phantom), 2 dimensions (ACR), and 3 dimensions (NEMA) for established 2D or advanced, high-resolution 3D PET-CT scanners that has not been previously demonstrated.

## Conclusions

Since the LV circumferential and longitudinal dimensions are large compared to scanner resolution, myocardial partial volume loss is due to the *single dimension* of LV wall thickness averaging 15 mm for diastole and systole, hence needing one-dimensional partial volume correction. Partial volume activity loss and corrections based on 2 dimensional ACR rods and 3 dimensional NEMA spheres overestimate PV correction for both Rb-82 and F-18 with corresponding erroneously high-quantitative perfusion by +50% to +150% compared to reference values validated clinically by outcomes data.^7^,^8^

Therefore, accurate PV loss and its correction are optimally derived from a one-dimensional phantom imaged with Rb-82 or F-18 to obtain the relative ratio of the 15mm/20mm wide single dimension target. Since ACR and NEMA phantoms are widely available, their overestimated PV loss and corrections for quantitative LV myocardial perfusion can be corrected to the needed one-dimensional partial volume correction with a simple equation applicable to all cardiac PET-CT scanners.

## Electronic supplementary material

Below is the link to the electronic supplementary material.

Electronic supplementary material 1 (PPTX 320 kb)

Electronic supplementary material 2 (M4A 2372 kb)

Electronic supplementary material 3 (DOCX 225 kb)

## Abbreviations

ACR: American College of Radiology
NEMA: National Electrical Manufactures Association
CAD: Coronary artery disease
CFC: Coronary flow capacity
KS: Kolmogorov–Smirnov statistic
LV: Left ventricule
MACE: Major adverse cardiovascular events (here death, MI, revascularization)
PET: Positron emission tomography
PV: Partial volume
ROI: Region of interest
