# Supplementary material for: Pitfalls in quantitative myocardial PET perfusion I: Myocardial partial volume correction
Source: J Nucl Cardiol. 2020 Feb 24;27(2):386–96. doi: 10.1007/s12350-020-02073-9 (PMC7174249; doi:10.1007/s12350-020-02073-9)
Supplement: Supplementary file 1 — Electronic supplementary material 1 (PPTX 320 kb) [file 12350_2020_2073_MOESM1_ESM.pptx]

## Slide 1
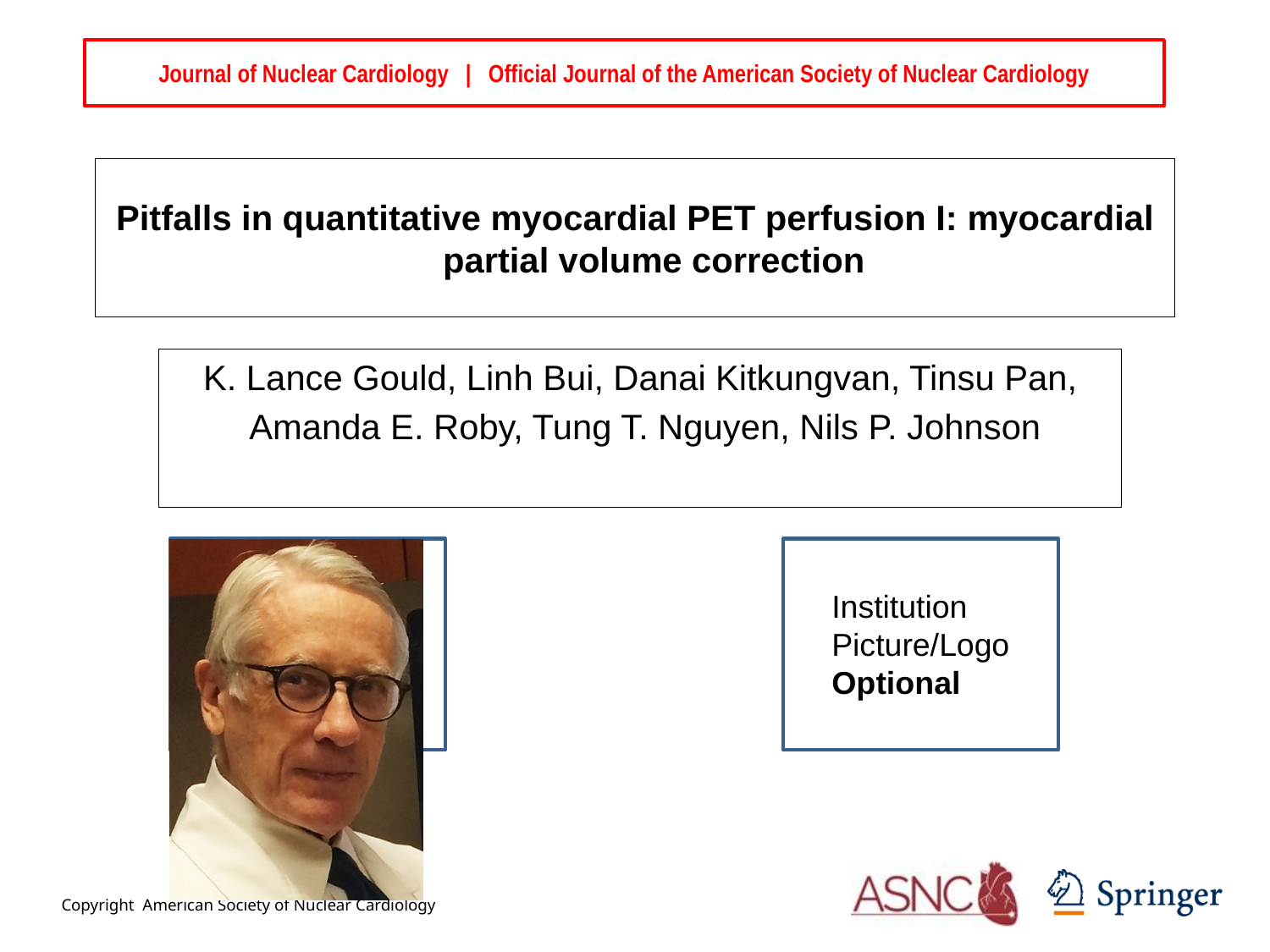

Journal of Nuclear Cardiology | Official Journal of the American Society of Nuclear Cardiology
# Pitfalls in quantitative myocardial PET perfusion I: myocardial partial volume correction
K. Lance Gould, Linh Bui, Danai Kitkungvan, Tinsu Pan,
 Amanda E. Roby, Tung T. Nguyen, Nils P. Johnson
Head shot of author
required
Institution
Picture/Logo
Optional
Copyright American Society of Nuclear Cardiology

## Slide 2
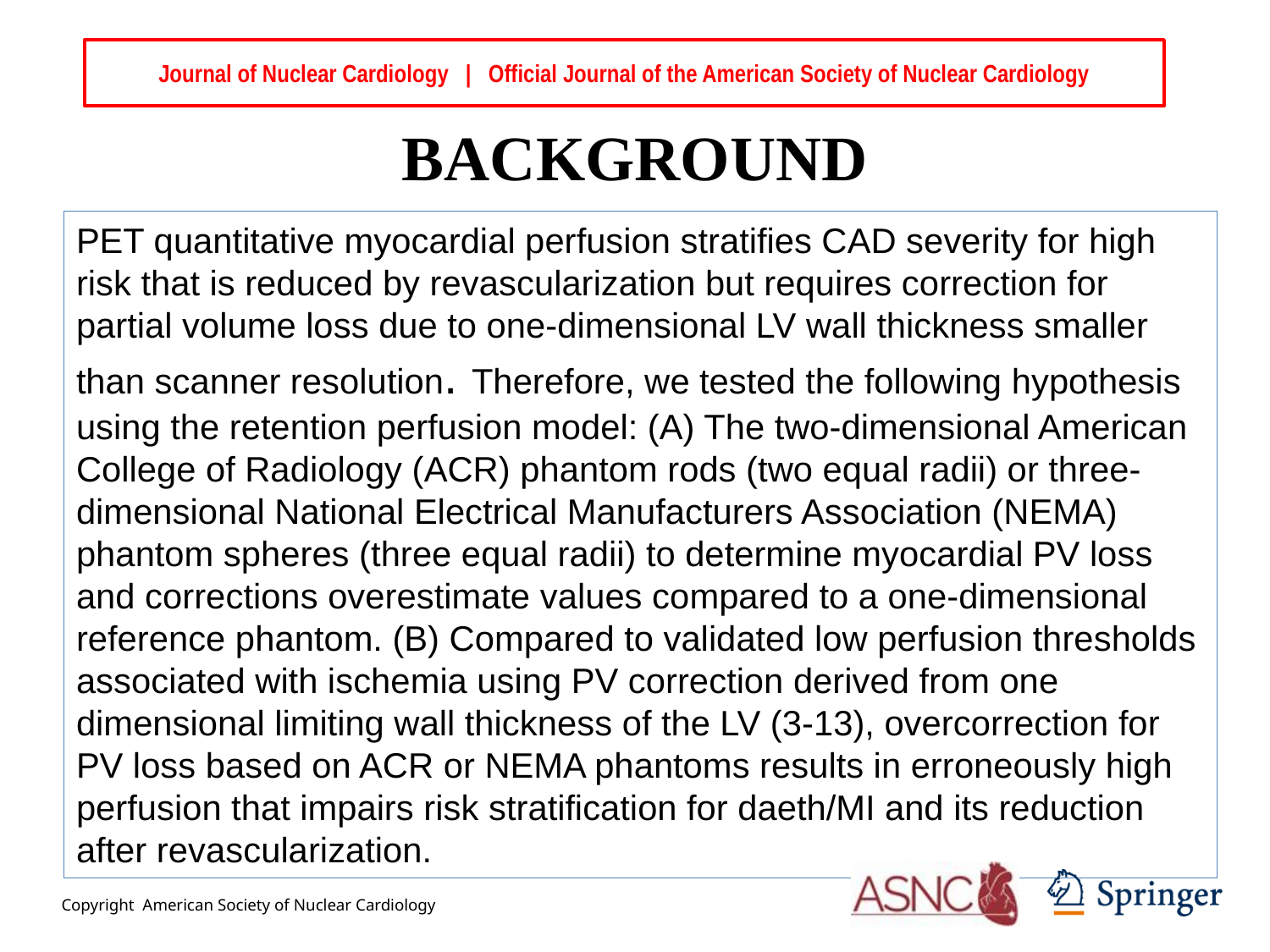

Journal of Nuclear Cardiology | Official Journal of the American Society of Nuclear Cardiology
# BACKGROUND
PET quantitative myocardial perfusion stratifies CAD severity for high risk that is reduced by revascularization but requires correction for partial volume loss due to one-dimensional LV wall thickness smaller than scanner resolution. Therefore, we tested the following hypothesis using the retention perfusion model: (A) The two-dimensional American College of Radiology (ACR) phantom rods (two equal radii) or three-dimensional National Electrical Manufacturers Association (NEMA) phantom spheres (three equal radii) to determine myocardial PV loss and corrections overestimate values compared to a one-dimensional reference phantom. (B) Compared to validated low perfusion thresholds associated with ischemia using PV correction derived from one dimensional limiting wall thickness of the LV (3-13), overcorrection for PV loss based on ACR or NEMA phantoms results in erroneously high perfusion that impairs risk stratification for daeth/MI and its reduction after revascularization.
Copyright American Society of Nuclear Cardiology

## Slide 3
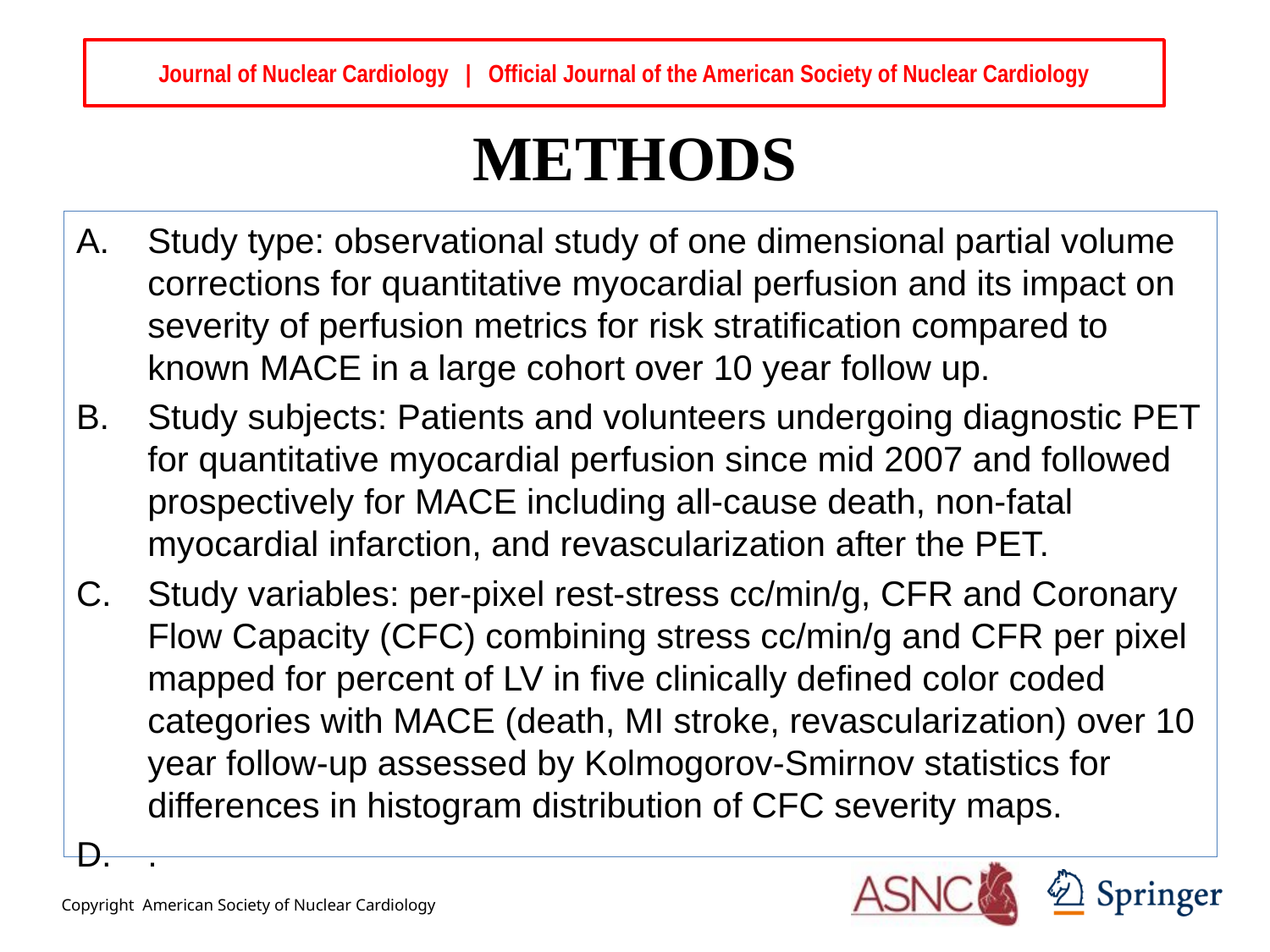

Journal of Nuclear Cardiology | Official Journal of the American Society of Nuclear Cardiology
# METHODS
Study type: observational study of one dimensional partial volume corrections for quantitative myocardial perfusion and its impact on severity of perfusion metrics for risk stratification compared to known MACE in a large cohort over 10 year follow up.
Study subjects: Patients and volunteers undergoing diagnostic PET for quantitative myocardial perfusion since mid 2007 and followed prospectively for MACE including all-cause death, non-fatal myocardial infarction, and revascularization after the PET.
Study variables: per-pixel rest-stress cc/min/g, CFR and Coronary Flow Capacity (CFC) combining stress cc/min/g and CFR per pixel mapped for percent of LV in five clinically defined color coded categories with MACE (death, MI stroke, revascularization) over 10 year follow-up assessed by Kolmogorov-Smirnov statistics for differences in histogram distribution of CFC severity maps.
.
Copyright American Society of Nuclear Cardiology

## Slide 4
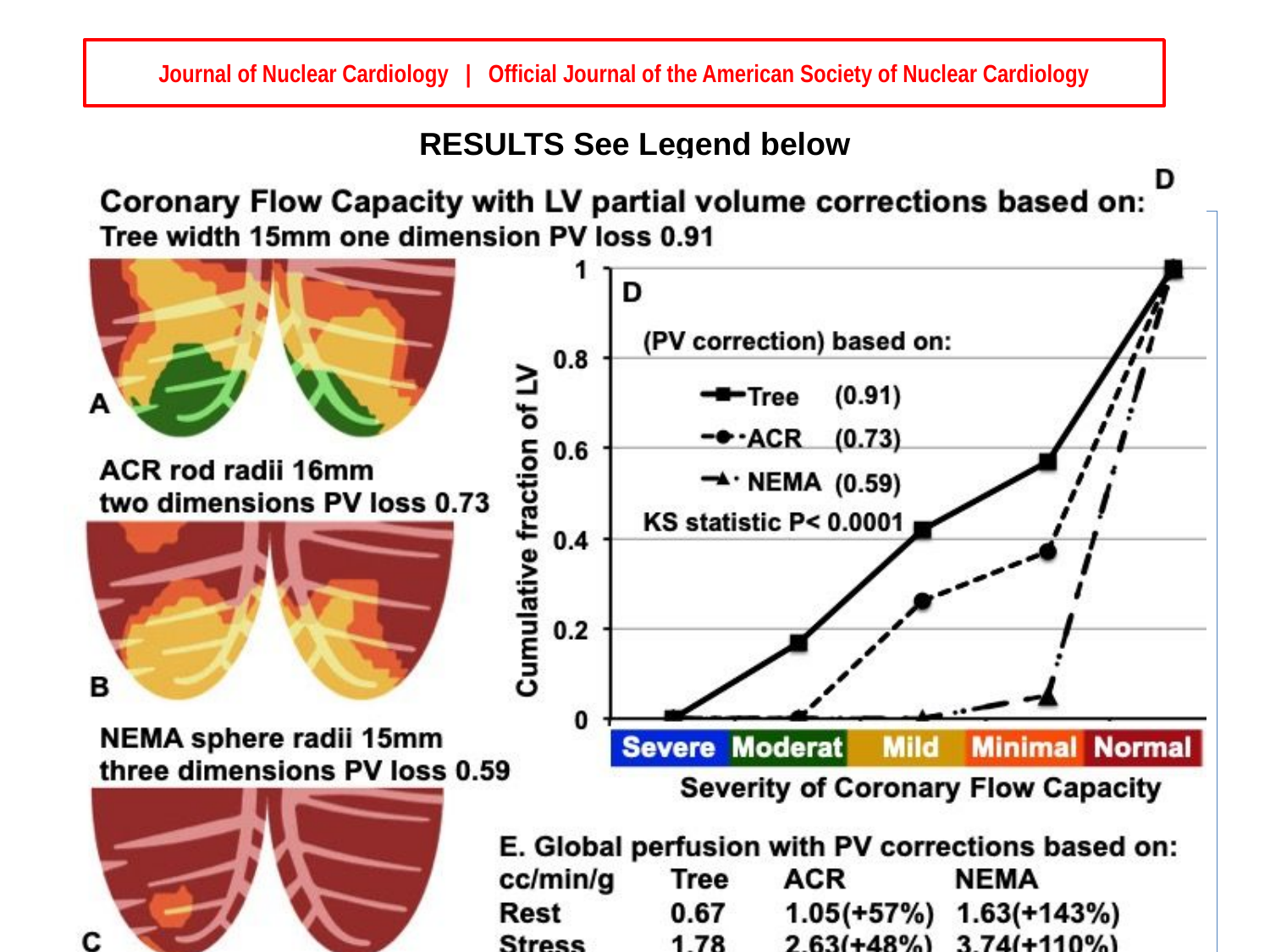

Journal of Nuclear Cardiology | Official Journal of the American Society of Nuclear Cardiology
# RESULTS See Legend below

## Slide 5
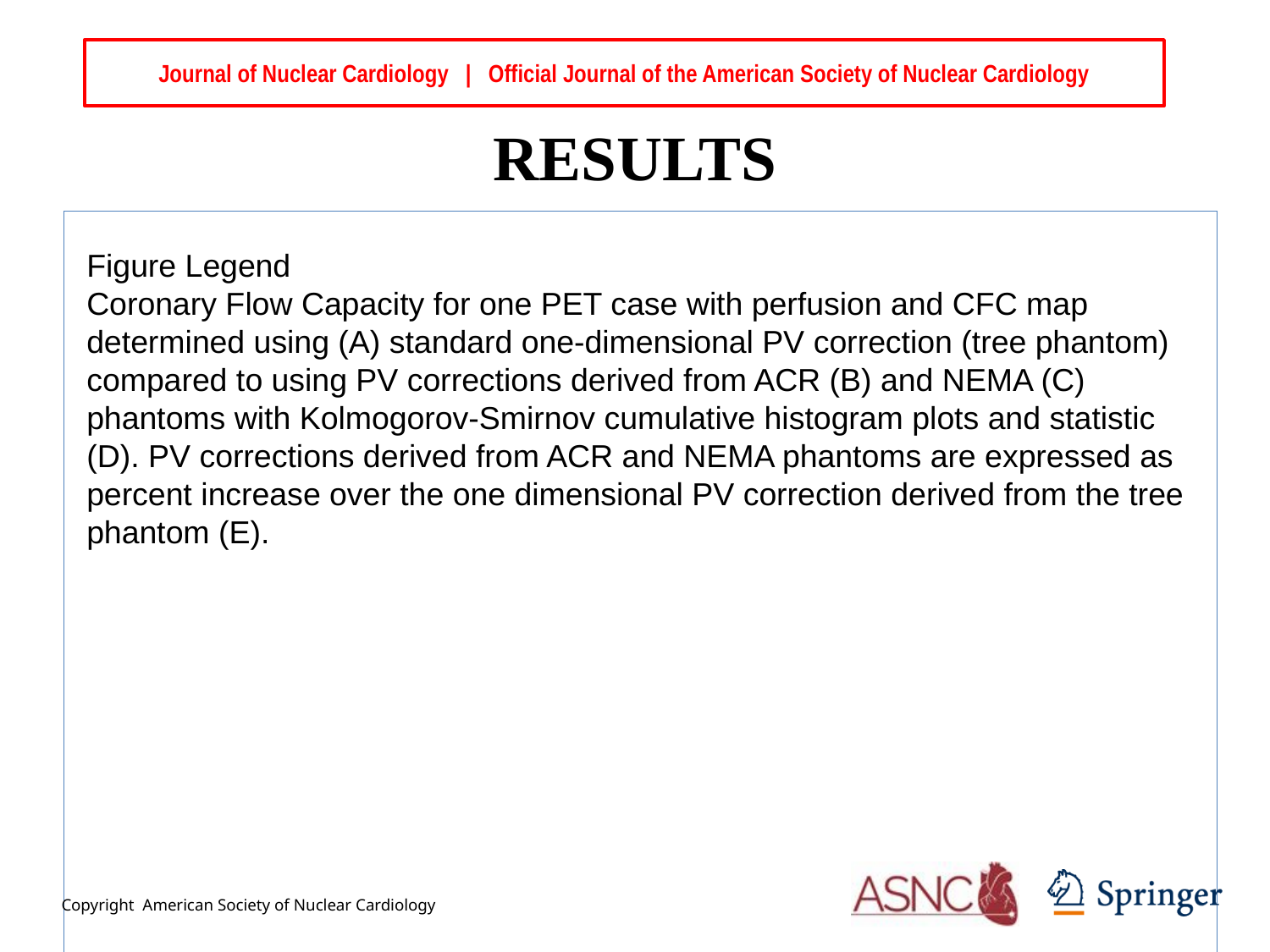

Journal of Nuclear Cardiology | Official Journal of the American Society of Nuclear Cardiology
# RESULTS
Figure Legend
Coronary Flow Capacity for one PET case with perfusion and CFC map determined using (A) standard one-dimensional PV correction (tree phantom) compared to using PV corrections derived from ACR (B) and NEMA (C) phantoms with Kolmogorov-Smirnov cumulative histogram plots and statistic (D). PV corrections derived from ACR and NEMA phantoms are expressed as percent increase over the one dimensional PV correction derived from the tree phantom (E).
Copyright American Society of Nuclear Cardiology

## Slide 6
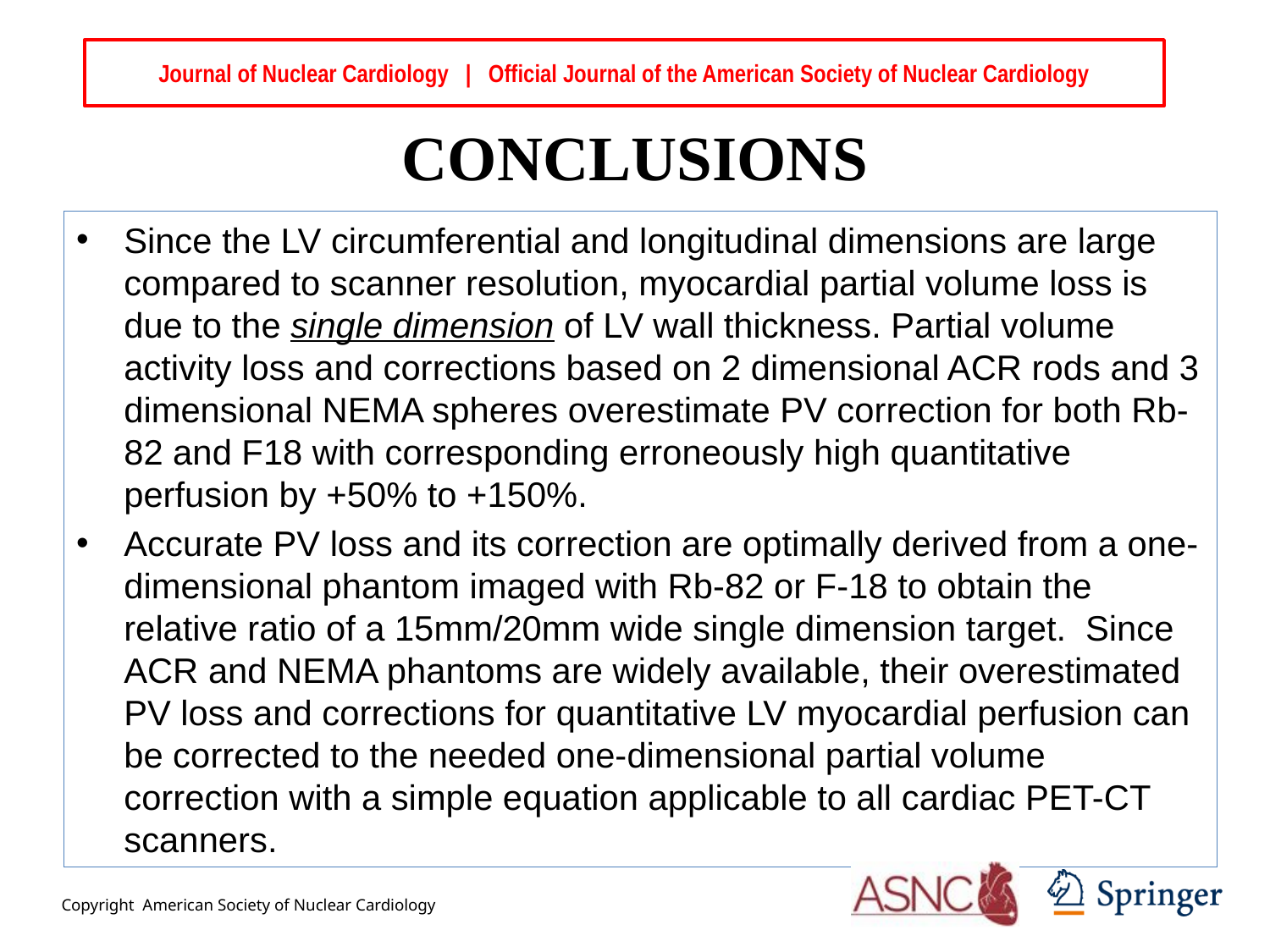

Journal of Nuclear Cardiology | Official Journal of the American Society of Nuclear Cardiology
# CONCLUSIONS
Since the LV circumferential and longitudinal dimensions are large compared to scanner resolution, myocardial partial volume loss is due to the single dimension of LV wall thickness. Partial volume activity loss and corrections based on 2 dimensional ACR rods and 3 dimensional NEMA spheres overestimate PV correction for both Rb-82 and F18 with corresponding erroneously high quantitative perfusion by +50% to +150%.
Accurate PV loss and its correction are optimally derived from a one-dimensional phantom imaged with Rb-82 or F-18 to obtain the relative ratio of a 15mm/20mm wide single dimension target. Since ACR and NEMA phantoms are widely available, their overestimated PV loss and corrections for quantitative LV myocardial perfusion can be corrected to the needed one-dimensional partial volume correction with a simple equation applicable to all cardiac PET-CT scanners.
Copyright American Society of Nuclear Cardiology
