# Supplementary material for: Pitfalls in quantitative myocardial PET perfusion I: Myocardial partial volume correction
Source: J Nucl Cardiol. 2020 Feb 24;27(2):386–96. doi: 10.1007/s12350-020-02073-9 (PMC7174249; doi:10.1007/s12350-020-02073-9)
Supplement: Supplementary file 3 — Electronic supplementary material 3 (DOCX 225 kb) [file 12350_2020_2073_MOESM3_ESM.docx]

**ON-LINE-RESOURCE-1 10-23-19**

Perfusion in cc/min/g for each of 64 radial pixels for each of 20 tomographic images are calculated by the following equation in the On-line Resource Equation OR1:

Equation OR1 P = M/[T**_m_**][1-e**^-(0.45+0.16P)/P^**][A][C**_A_**][C**_M_**]

Where A is decay the corrected integrated time activity in an ROI located at the peak activity in the left atrium or aortic root on the high quality early 2 minute image.  M is decay corrected myocardial uptake image divided by its time interval T**_m_** of acquisition as the "instantaneous" activity of myocardial Rb-82 that is "flat" during the 5 minute myocardial image due to trapped Rb-82 retained without cellular leaking out from viable non-infarcted myocardium. C**_A_** is the partial volume correction of 1.0 for integrated time activity of the left atrium or aortic root both being > 1.5cm.  C**_M_** is the myocardial partial volume correction of 0.9 in the denominator for heart rate dependent, diastolic-systolic varying wall thickness as previously published for the GE DST-16 PET-CT. Density of myocardium is 1.05gm/cc or 0.95cc/gm. The [brackets] term accounts for experimentally determined relation of decreasing Rb-82 myocardial trapping as perfusion increases. The pixel perfusion data then undergos a 5x5 moving smooth.

**Coronary Flow Capacity Map**

**
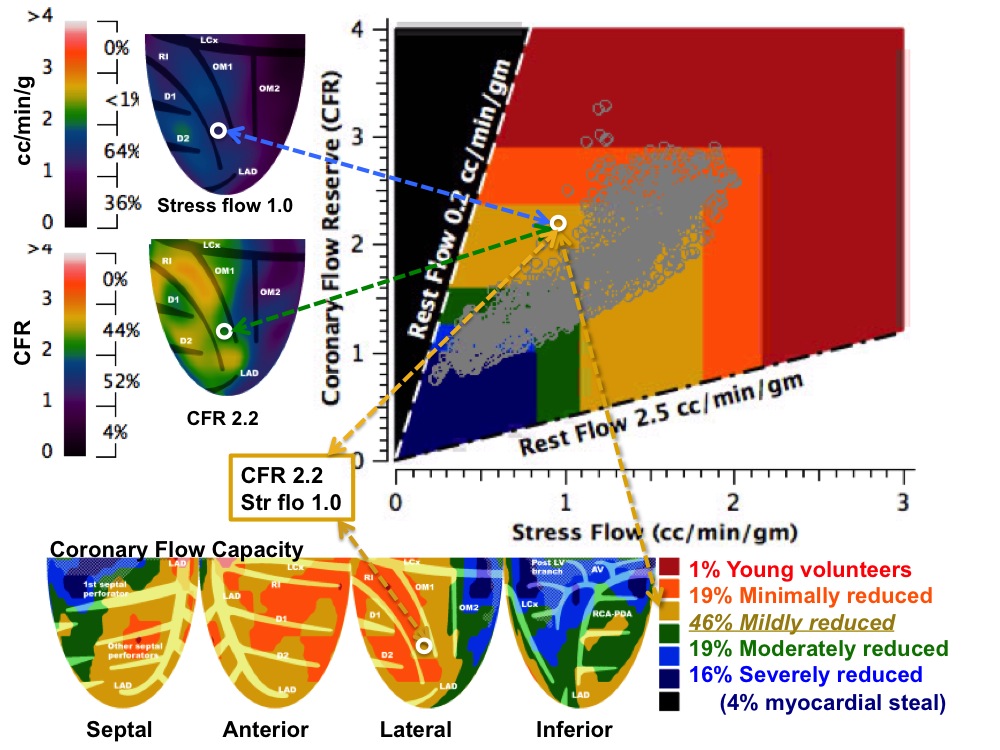
**

**Supplement Figure Legend.** Schema for color coding each pixel of the Coronary Flow Capacity Map (CFC). As previously reported (4-6), the color coded ranges of Coronary Flow Reserve (CFR) and stress perfusion in cc/mi/g of the CFC map are based on objective, predefined clinical criteria into the clinically groups detailed below. The perfusion boundaries between clinical groups were determined by objective ROC analysis for optimum CFR and stress perfusion separating the predefined clinical groups as follows:

Healthy Young Volunteers < 40 years old color coded red (Normal) with CFR > 2.9 or stress flow > 2.17 cc/min/g down to the upper boundary of Minimally reduced CFC below.

Risk factors only with no documented or known CAD color-coded orange (Minimally reduced CFC) with CFR ≤ 2.9 and stress flow ≤ 2.17 cc/min/g down to the upper boundary of the Mildly reduced CFC below.

Coronary Artery Disease (CAD) by past myocardial infarction (MI), abnormal angiogram or revascularization without angina or ECG changes during dipyridamole stress color coded yellow (Mildly reduced CFC) with CFR ≤ 2.38 and stress flow ≤ 1.82 cc/min/g down to the upper boundary of the Moderately reduced CFC below.

Moderately Reduced CFC with dipyridamole induced relative stress defect or angina or > 1mm ST depression (one of three criteria) color coded green (Moderately reduced CFC) with CFR ≤ 1.6 and stress flow ≤ 1.1 cc/min/g down to the upper boundary of the Severely reduced CFC below.

Severely Reduced CFC with dipyridamole induced definite ischemia with angina or > 1mm ST depression or a quadrant stress defect of ≤ 60% of maximum relative activity stress (two or more of three criteria) color coded blue (Severely reduced CFC) with CFR ≤ 1.27 and stress perfusion ≤ 0.83 (called CFCsevere). Myocardial Steal is defines as CFR < 1.0 color-coded dark blue.

**A procedure for determining the partial volume correction for LV wall thickness for quantitative myocardial perfusion by PET.**

**Rationale**

Since the LV circumferential and longitudinal dimensions are large compared to scanner resolution, the entire LV myocardial partial volume loss is due to the *single dimension* of LV wall thickness averaging 15mm thence needing one dimensional partial volume correction. Therefore, accurate PV loss and its correction is optimally derived from the one dimensional Tree phantom imaged with Rb-82 or F18 to obtain the relative ratio of the 15mm/20mm wide Tree target yielding a PV correction of 0.90 for Rb-82 with the GE DST 16 PET-CT (Table 1).

Since the Tree phantom is not commercially available, PV correction for LV can be derived from imaging the two dimensional ACR phantom with two equal radii as the relative ratio 16mm/25mm rods that approximates the correct one-dimensional PV loss and correction of 0.90 for LV wall thickness for Rb-82. The three dimensional NEMA spheres with three equal radii substantially over estimates the PV correction for both Rb-82 and F18 with corresponding perfusion in cc/min/g over estimated by 50% to 150% over the one dimensional PV correction needed for the LV. Overestimated PV corrections, rest-stress perfusion and CFC arising from inappropriate use of ACR and NEMA phantoms causes substantial erroneous prediction of CV risk or risk stratification with potentially adverse impact on PET guided management of CAD.

Therefore, for cardiac PET, the optimal LV myocardial partial volume correction is derived as the relative ratio of the 15mm/25mm ACR rods filled with F18 at a concentration of 10µCi/ml that is the myocardial maximum observed clinically. The aortic and LA target sizes for arterial input are over 20mm and therefore do not require PV correction.

**Procedure**

For optimal quantitative perfusion and CFC, associated MACE and outcomes after CFC guided revascularizarion based on 6000 PETs, cardiac PET-CT scanners should be tested as follows:

1. In 300cc of water in a beaker, add 3 mCi of F18 for a concentration of 10µCi/ml.

2. Fill the 16mm and 25mm diameter rods of the ACR phantom with F18 and image in 2D or 3D mode with rod length perpendicular to the xy plane of the detectors and parallel to the z axis or long axis of the scanner bed. The partial volume activity loss is the relative activity ratio of the ACR 16/25 mm diameter rods that is also the partial volume correction entered into the partial volume option that inserts the value in the denominator of the HeartSee flow model equations.

3. Use reconstruction and filter settings that provide minimal clinically useful smoothing with the smallest PV loss of approximately 0.0.85 for F18 in the16/25mm diameter rods of the ACR phantoms corrected by the equation below for Rb-82 to 0.90 (Table 1). Enter the corresponding measured PV correction in the range of 0.90 for Rb-82 or 0.94 for F-18 into the software window for correcting the myocardial activity. Since the aorta and left atrium are greater than 20mm, PV correction for arterial input from aorta or LA is not needed.

4. As clinical confirmation, image 5 healthy young volunteers less than 40 years old with no medical problems, on no meds (particularly no ADHD drugs that block vasodilator stress), off caffeine for 24 or preferably 36 hours. Perfusion at rest should be 0.5 to 0.8 cc/min/g and at stress ≥ 210 cc/min/g with CFR 3.8 or greater and therefore can be referenced to HeartSee data base for outcomes as approved by the FDA (510 K171303).

The optimal one-dimensional PV correction for LV wall thickness can be derived from the three dimensional NEMA phantom spheres with three equal radii using the following Equation of online resource 1:

Online Resource Equation 1 PV1D = (PV**_observ_ /***R***^n^** )**^1/n^** = **^n^**√(PV**_observ_** /*R***^n^**)

where n is the square root for ACR and cube root for NEMA observed or measured activity recovery for the 16mm/25mm ACR rods and 17/28mm NEMA spheres.

With this equation, the appropriate one dimensional PV correction can be determined for either Rb or F18 in the 15mm 1-D Tree width, the 16mm 2-D ACR rods or the 17mm 3-D NEMA spheres. For F18, the R term is 1.0 and for Rb, the *R* term is the relative activity recovery of Rb/F18 from Table 1, 0.96 (0.90/0.94).

For example, for an observed activity recovery of 0.72 from the 17mm/28mm NEMA spheres, the PV correction for the one dimensional PV correction for cardiac imaging with F18 is calculated as:

PVD1 = (0.72/1)**^1/3^** or **^3^**√0.72 = 0.90 that approximates with the directly measured activity recovery of 0.94 for F18 from the 15mm/20mm activity ratio of the Tree width in Table 1.

For an observed activity recovery of 0.59 from the 17mm/28mm NEMA spheres, the PV correction for the one dimensional PV correction for cardiac imaging with Rb is calculated as:

PVD1 = (0.59/0.96**^3^**)**^1/3^** or **^3^**√0.67 = 0.87 that compares with the directly measured activity recovery of 0.9 for Rb from the 15mm/20mm activity ratio of the Tree width from Table 1.
